# Layered composites of PEDOT/PSS/nanoparticles and PEDOT/PSS/phthalocyanines as electron mediators for sensors and biosensors

**DOI:** 10.3762/bjnano.7.186

**Published:** 2016-12-08

**Authors:** Celia García-Hernández, Cristina García-Cabezón, Fernando Martín-Pedrosa, José Antonio De Saja, María Luz Rodríguez-Méndez

**Affiliations:** 1 Grupo Uvasens, Escuela de Ingenierías Industriales, Universidad de Valladolid, Paseo del Cauce 59, 47011 Valladolid, Spain

**Keywords:** catechol, gold nanoparticle hydroquinone, PEDOT/PSS, phthalocyanine, voltammetric sensor

## Abstract

The sensing properties of electrodes chemically modified with PEDOT/PSS towards catechol and hydroquinone sensing have been successfully improved by combining layers of PEDOT/PSS with layers of a secondary electrocatalytic material such as gold nanoparticles (PEDOT/PSS/AuNPs), copper phthalocyanine (PEDOT/PSS/CuPc) or lutetium bisphthalocyanine (PEDOT/PSS/LuPc_2_). Layered composites exhibit synergistic effects that strongly enhance the electrocatalytic activity as indicated by the increase in intensity and the shift of the redox peaks to lower potentials. A remarkable improvement has been achieved using PEDOT/PSS/LuPc_2_, which exhibits excellent electrocatalytic activity towards the oxidation of catechol. The kinetic studies demonstrated diffusion-controlled processes at the electrode surfaces. The kinetic parameters such as Tafel slopes and charge transfer coefficient (α) confirm the improved electrocatalytic activity of the layered electron mediators. The peak currents increased linearly with concentration of catechol and hydroquinone over the range of 1.5 × 10^−4^ to 4.0 × 10^−6^ mol·L^−1^ with a limit of detection on the scale of μmol·L^−1^. The layered composite hybrid systems were also found to be excellent electron mediators in biosensors containing tyrosinase and laccase, and they combine the recognition and biocatalytic properties of biomolecules with the unique catalytic features of composite materials. The observed increase in the intensity of the responses allowed detection limits of 1 × 10^−7^ mol·L^−1^ to be attained.

## Introduction

The assessment of phenols has been successfully achieved using electrodes chemically modified with a variety of materials [1–10]. In addition, some mixtures of electrocatalytic materials such as polyaniline or polypyrrole with graphene can enhance the sensitivity of the sensors while lowering the detection limits [11–13].

Poly(3,4-ethylene dioxythiophene)/poly(styrene sulfonic acid) (PEDOT/PSS) is an appealing electrocatalytic material due to its high conductivity and low redox potential. PEDOT/PSS is soluble in water and the polymer can be mixed with water-soluble electrocatalytic materials or colloidal metal nanoparticles providing an excellent method to modulate the sensing properties by means of a synergistic effect [14–15]. However, a large number of electrocatalytic materials are soluble in organic solvents and this fact limits the number of electrocatalytic materials that can be used to modulate the PEDOT/PSS properties. One possible strategy to avoid this problem is to develop layered composites where a film of a secondary electrocatalytic material is deposited on top of a PEDOT/PSS layer.

The first objective of this work was to develop novel electrochemical sensors based on layered composites formed by alternating layers of PEDOT/PSS and a secondary electrocatalytic material (EM) (PEDOT/PSS/EM) and to evaluate the existence of synergistic effects. As EMs, three different materials with different characteristics and electrocatalytic activity towards phenols were tested, including gold nanoparticles (AuNPs), a copper phthalocyanine (CuPc) (a p-type semiconductor) and a lutetium bisphthalocyanine (LuPc_2_) (a sandwich-type derivative with free radical character which is an intrinsic semiconductor) [9,16–18].

On the other hand, enzymatic electrochemical biosensors based on phenol oxidases are a good alternative to analyze phenols due to their high sensitivity and selectivity. Tyrosinase oxidizes monophenols and o-diphenols to the corresponding quinone, whereas laccase catalyzes the oxidation of a larger variety of aromatic compounds such as substituted mono- and poly-phenols, with subsequent formation of radicals, which are converted to quinones in the second stage of the oxidation [19]. Tyrosinase (Tyr) and laccase (Lac) must be combined with electron mediators to facilitate the transfer of electrons from the enzyme to the electrode [20]. PEDOT/PSS is becoming popular as an electron mediator in biosensing [21–22]. Gold nanoparticles and phthalocyanines have also been positively demonstrated as electron mediators in tyrosinase biosensors [16,23–26]. Also, in the case of biosensors, an improvement of the electron mediator activity could be expected by using layered composites formed by two electron mediators. For this reason, the second objective of this work was to develop phenol biosensors containing Tyr or Lac using layered composites of PEDOT/PSS and AuNPs, CuPc or LuPc_2_ as electron mediators and to investigate the existence of synergistic effects in the electron transfer occurring during biosensing.

In order to pursue the two objectives proposed, PEDOT/PSS/EM electrodes were prepared by depositing a layer of AuNPs, CoPc or LuPc_2_ on the top of the PEDOT/PSS film by means of spin coating to obtain PEDOT/PSS/AuNP, PEDOT/PSS/CuPc and PEDOT/PSS/LuPc_2_ sensors, respectively. The electrodes were used as the working electrodes in cyclic voltammetry. The sensing properties and any synergistic effects in the layered composite electrodes were evaluated towards catechol (1,2-dihydroxybenzene) and hydroquinone (1,4-dihydroxybenzene) which are two dihydroxybenzene isomers with many ubiquitous industrial applications [27–28]. Additionally, the kinetic parameters and limit of detection were calculated.

Further studies were carried out using the modified electrodes combined with tyrosinase (PEDOT/PSS/EM-Tyr) and laccase (PEDOT/PSS/EM-Lac) enzymes. The electron mediator properties of the layered composites with respect to the performance of biosensors containing tyrosinase or laccase were evaluated.

## Results and Discussion

### Structure and conductivity of the PEDOT/PSS/EM sensors

**Figure 1:**
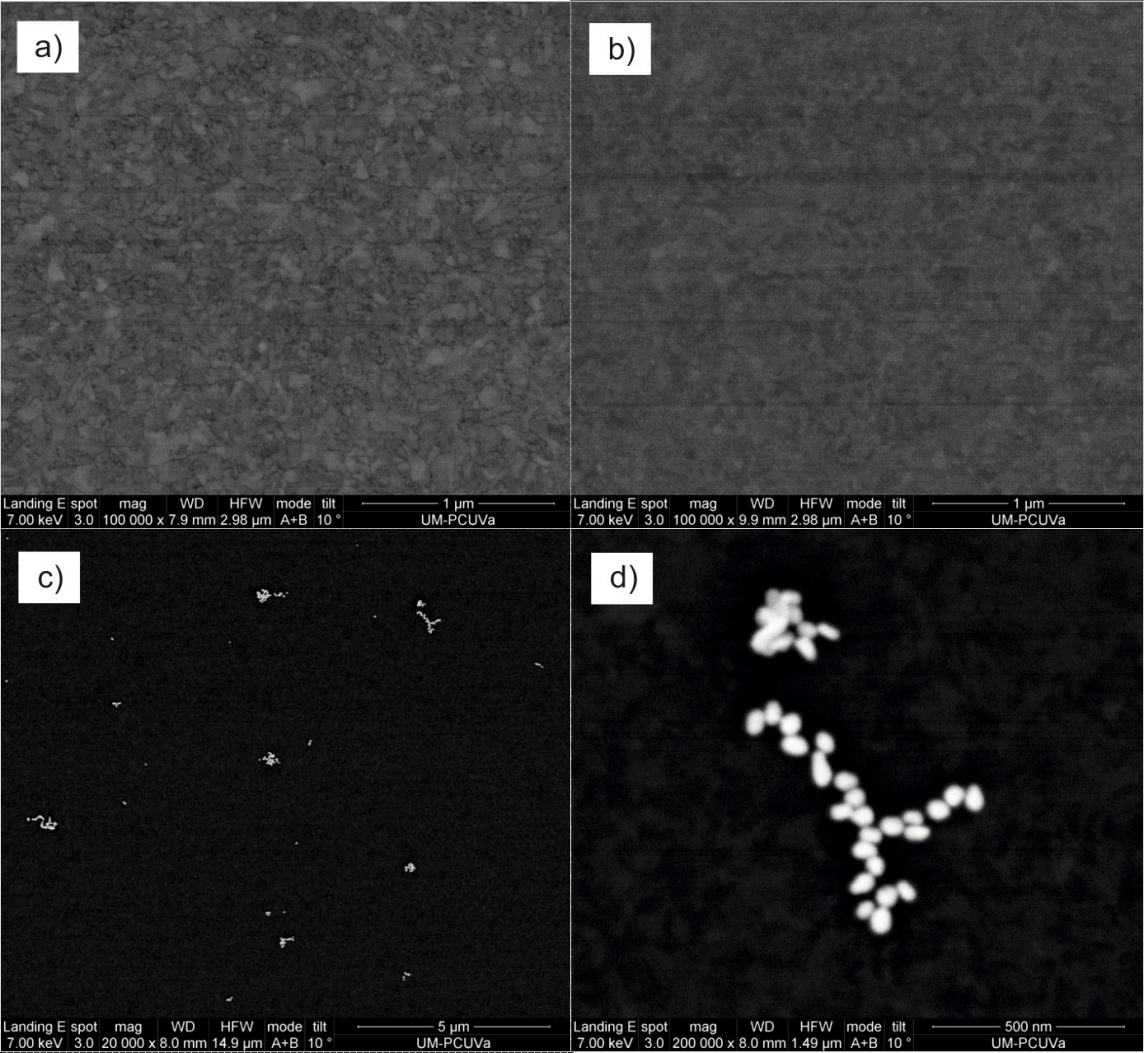
SEM images of electrodes modified with a) PEDOT/PSS, b) PEDOT/PSS/CuPc, c) PEDOT/PSS/AuNP and d) PEDOT/PSS/AuNP (higher magnification).

ITO electrodes were modified by depositing a PEDOT/PSS layer by means of spin coating. Layered composite electrodes were prepared by depositing layers of a secondary electrocatalytic material (AuNPs, CoPc or LuPc_2_) on top of the PEDOT/PSS film to obtain PEDOT/PSS/AuNP, PEDOT/PSS/CuPc and PEDOT/PSS/LuPc_2_, respectively. The surface morphologies of the modified electrodes were analyzed by SEM. As observed in Figure 1, the PEDOT/PSS film showed a regular, smooth and homogeneous structure. Further modification with CuPc and LuPc_2_ did not affect the morphology. SEM images of the PEDOT/PSS/AuNP electrode confirmed that small clusters of 40–60 nm diameter AuNPs were homogeneously distributed on the surface of the film. The increase in the effective surface area due to the AuNPs could be highly beneficial for electrochemical applications in sensors.

The square resistance and resistivity were measured with the four-point probe test (Table 1). The current–voltage curves obtained from PEDOT/PSS and PEDOT/PSS/EM electrodes exhibited a good linear fit with correlation coefficients higher than 0.999.

**Table 1:** Square resistance (*R*_sq_) and conductivity (ρ) of the PEDOT/PSS and PEDOT/PSS/EM electrodes.

	*R*_sq_ (Ω·sq^−1^)	ρ (×10^−6^ Ω·m)

PEDOT/PSS	13.9	27.9
PEDOT/PSS/LuPc_2_	6.4	15.7
PEDOT/PSS/CuPc	9.6	22.4
PEDOT/PSS/AuNP	7.9	19.7

It has been reported that the addition of different dopants to an aqueous solution of PEDOT/PSS produces an enhancement in the conductivity of PEDOT/PSS films obtained from the solution [29–30]. In our case, the additives were deposited as a thin film on the top of the PEDOT/PSS layer and the same effect was observed. Layered composites exhibited better conductivity than PEDOT/PSS following the order PEDOT/PSS/LuPc_2_ > PEDOT/PSS/AuNPs > PEDOT/PSS/CuPc. This enhanced conductivity can contribute to the observed improvement of the electron transfer rate of the sensors that will be shown in the following.

The mechanism of the conductivity enhancement can be different depending on the material deposited on the PEDOT/PSS layer [29–31]. In the case of CuPc or AuNPs, the improvement in the conductance can be due to the increase in the charge carrier mobility and/or in the large effective surface provided by the metallic AuNPs. In the case of LuPc_2_, which is an intrinsic semiconductor and has a free radical character [32], the enhancement can be due to the increase in charge carriers produced by the interaction between the PEDOT/PSS chains and the free carriers in LuPc_2_.

### Electrochemical characterization of the PEDOT/PSS/EM sensors

**Figure 2:**
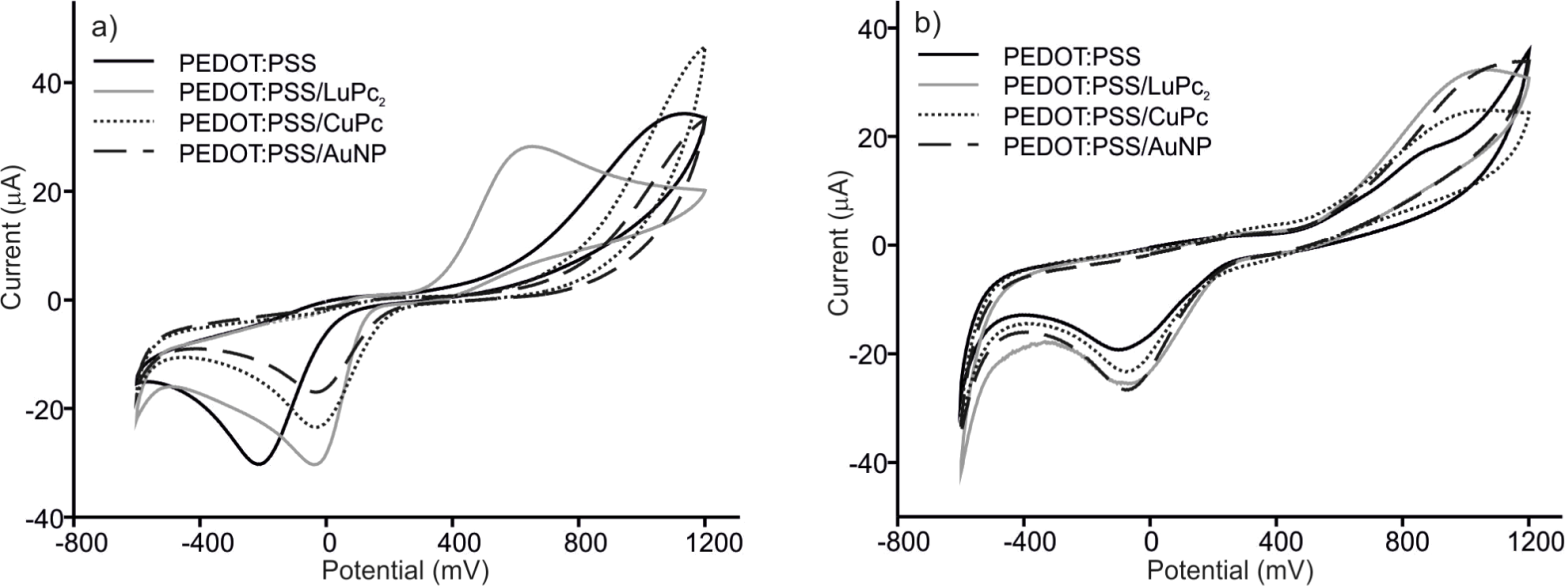
Cyclic voltammograms of PEDOT/PSS, PEDOT/PSS/LuPc_2_, PEDOT/PSS/CoPc and PEDOT/PSS/AuNP sensors in (a) catechol and (b) hydroquinone 1.5 × 10^−4^ mol·L^−1^ with 0.01 mol·L^−1^ phosphate buffer as the supporting electrolyte. Scan rate 0.1 V·s^−1^.

The electrochemical characteristics of PEDOT/PSS/EM electrodes were analyzed using cyclic voltammetry. Voltammograms of PEDOT/PSS, PEDOT/PSS/AuNPs, PEDOT/PSS/CuPc and PEDOT/PSS/LuPc_2_ electrodes immersed in catechol and hydroquinone 1.5 × 10^−4^ mol·L^−1^ with 0.01 mol·L^−1^ phosphate buffer as the supporting electrolyte are shown in Figure 2. The response of ITO glass is not shown in the figure due to the low intensity, characterized by one cathodic peak at −0.4 V. At positive potentials, the oxidation of phenols could not be observed because it occurs at potentials higher than the working range.

In the case of PEDOT/PSS modified electrodes, catechol and hydroquinone were oxidized at the working electrode to form 1,2-benzoquinone and 1,4-benzoquinone, respectively. During the reaction, oxygen and hydrogen also form H_2_O_2_. During the reverse scan, quinones were reduced to the phenolic compound.

PEDOT/PSS and PEDOT/PSS/EM electrodes showed much larger current response (one order of magnitude) towards catechol than bare ITO glass. In addition, the presence of nanoparticles or phthalocyanines produced a shift of the cathodic wave to lower potentials (−0.4 V in unmodified ITO, −0.2 V in PEDOT/PSS and −0.01 V in PEDOT/PSS/EM). The electrocatalytic effect was stronger on the PEDOT/PSS/LuPc_2_ electrode were a drastic decrease in the oxidation potential (from 1 V in PEDOT/PSS to 0.65 V in PEDOT/PSS/LuPc_2_) was observed. The improvement of the performance observed in PEDOT/PSS/EM, where two electrocatalytic materials are combined, can be attributed to the enhanced electron transfer rate provided by the composites due to the interactions between two electrocatalytic components. In particular, the excellent response of the PEDOT/PSS/LuPc_2_ electrode can be due to the interaction between the free radical LuPc_2_ and the radical intermediate produced during the oxidation of catechol. In addition, the aromatic structure of catechol can also establish strong π–π interactions with phthalocyanines, thus facilitating the electron transfer.

The interaction of catechol and hydroquinone with electrodes was similar. However, a clear difference was observed with PEDOT/PSS/LuPc_2_ electrodes where a strong decrease in the oxidation peak voltage was not observed [33].

**Figure 3:**
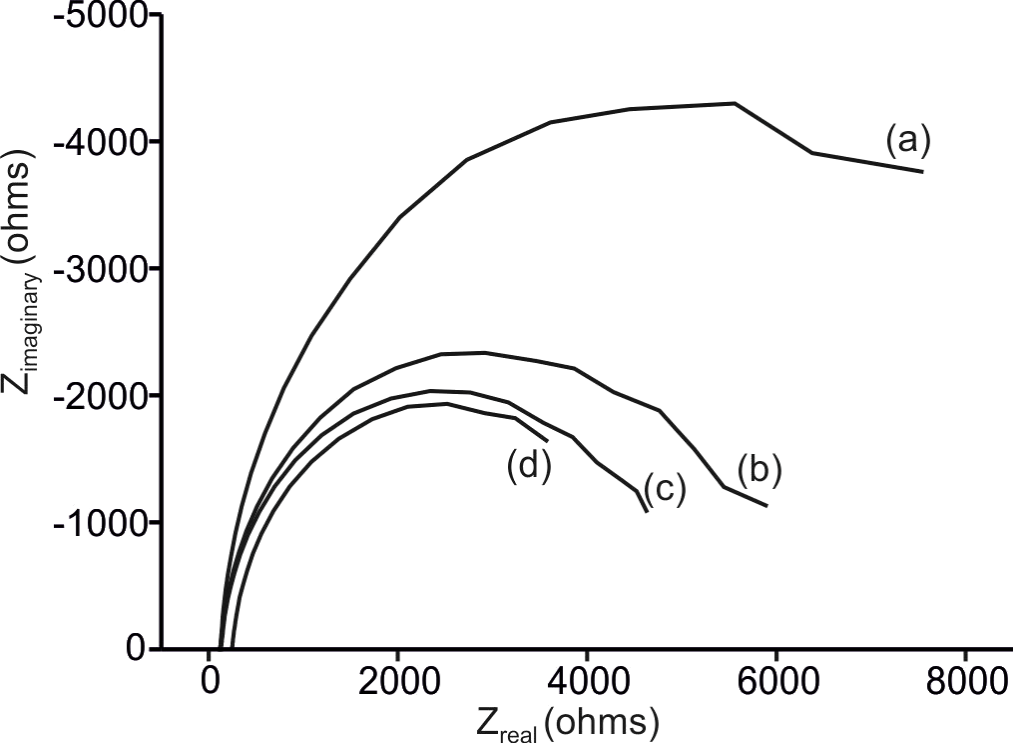
Nyquist plots collected at −0.5 V using (a) PEDOT/PSS; (b) PEDOT/PSS/CuPc; (c) PEDOT/PSS/LuPc_2_; and (d) PEDOT/PSS/AuNP. Electrodes were immersed in catechol 10^−3^ mol·L^−1^ with 0.01 mol·L^−1^ phosphate buffer (pH 7.0) as the supporting electrolyte. The frequency was swept logarithmically from 10^−2^ to 10^5^ Hz.

The electrocatalytic properties of the layered composite PEDOT/PSS/EM electrodes were further investigated using electrochemical impedance spectroscopy (EIS). At −0.5 V, a RMS sine wave was applied with frequencies varying logarithmically from 10^−2^ to 10^5^ Hz. Typical Nyquist plots obtained from a 10^−3^ mol·L^−1^ catechol solution are displayed in Figure 3. *R*_s_ represents the solution resistance. The semicircular part of the diagram at high frequencies corresponds to electron-transfer-limited processes, and the diameter is equivalent to the electron transfer resistance (*R*_ct_).

The impedance parameters were derived using a Randels equivalent circuit. The whole interface impedance was modelled by a constant phase element (CPE) (Equation 1 and Table 2):

[1]
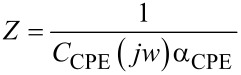


where *C*_CPE_ and α_CPE_ are the capacitance and coefficient of the constant phase element, respectively.

**Table 2:** Results obtained from the impedance spectroscopy measurements.

	*R*_s_ (Ω)	*C*_CPE_ (F)	α_CPE_	*R*_ct_ (Ω)

PEDOT/PSS	170.8	1.70 × 10^−5^	0.91	11430
PEDOT/PSS/LuPc_2_	127.4	2.23 × 10^−5^	0.92	4734
PEDOT/PSS/CuPc	120.3	2.62 × 10^−5^	0.92	5375
PEDOT/PSS/AuNPs	148.4	3.2 × 10^−5^	0.93	4346

Layered composites showed low *R*_ct_ values (≈4300–5300 Ω) which is about half of the *R*_ct_ observed in PEDOT/PSS (11500 Ω), indicating that charge transfer rates are higher in PEDOT/PSS/EM sensors. This agrees well with the aforementioned resistivity measurements. The smaller *R*_ct_ values revealed a low electron-transfer resistance which might be caused by the synergetic enhanced effect between PEDOT/PSS and the secondary EM.

### Electrochemical characterization of PEDOT/PSS/EM enzymatic biosensors

PEDOT/PSS/EM composites were also used as electron mediators in tyrosinase (PEDOT/PSS/EM-Tyr) and laccase-based (PEDOT/PSS/EM-Lac) biosensors. As tyrosinase is selective to the oxidation of o-diphenols and laccase catalyzes the oxidation of a larger variety of polyphenols, PEDOT/PSS/EM-Tyr sensors were used to detect catechol and PEDOT/PSS/EM-Lac sensors were used to detect hydroquinone.

**Figure 4:**
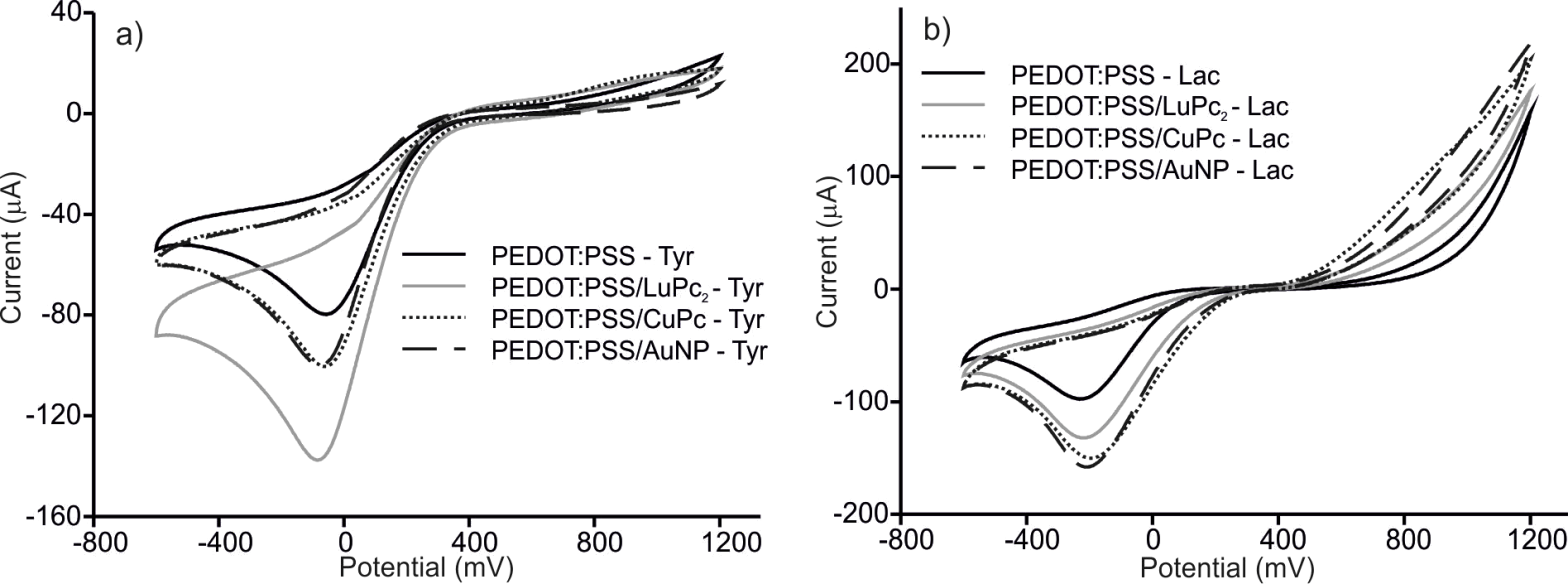
Cyclic voltammograms of (a) PEDOT/PSS/EM-Tyr immersed in catechol 1.5 × 10^−4^ mol·L^−1^and (b) PEDOT/PSS/EM-Lac immersed in hydroquinone 1.5 × 10^−4^ mol·L^−1^. Scan rate 0.1 V·s^−1^.

In PEDOT/PSS/EM-Tyr biosensors immersed in catechol, a drastic increase in intensity of the reduction peak at −0.05 V was observed (Figure 4). This increase is due to the simultaneous reduction of o-quinone formed by the electrochemical oxidation at positive potentials plus the reduction of the o-quinone formed by enzymatic oxidation. The electron transfer was promoted in the presence of PEDOT/PSS and further improved in the presence of a secondary electron mediator at PEDOT/PSS/EM electrodes. The presence of LuPc_2_ produced the largest increase in the peak intensity (−30 μA in PEDOT/PSS, −80 μA in PEDOT/PSS-Tyr and −132 μA in PEDOT/PSS/LuPc_2_-Tyr). No amplification was observed in the oxidation peak and the current showed the same values as those observed in nonenzymatic sensors (due to the increase in the scale, oxidation peaks cannot be clearly seen in the Figure). When biosensors containing laccase were immersed in the hydroquinone solution, a drastic increase in intensity of the reduction peak at −0.2 V was also observed (−20 μA in PEDOT/PSS, −85 μA in PEDOT/PSS-Lac and −130 μA in PEDOT/PSS/LuPc_2_-Lac). In this case, the intensity of the signal at positive potentials was also amplified. The results demonstrate that the layered composites developed in this work facilitate the electron transfer and the synergistic effect between the components was evidenced.

### Repeatability and reproducibility

Repetitive measurements were carried out in 10^−3^ mol·L^−1^ solutions to study the repeatability of the voltammograms. The results of five consecutive measurements showed a coefficient of variation lower than 2% in all cases. Additionally, the reproducibility of the electrodes was examined by cycling the electrodes in 10^−3^ mol·L^−1^ solutions using three electrodes prepared using the same method. The coefficient of variation in both cathodic and anodic peaks were found to be less than 4%, confirming the reproducibility of the method.

It is worth mentioning that the PEDOT/PSS/EM electrodes could be used up to 50 times with a decrease in the intensity of less than 5%. The PEDOT/PSS/EM enzymatic electrodes were found to be highly stable for ten scans. After that their functionality progressively decreased and could no longer be used.

### Scan rate dependence study

**Figure 5:**
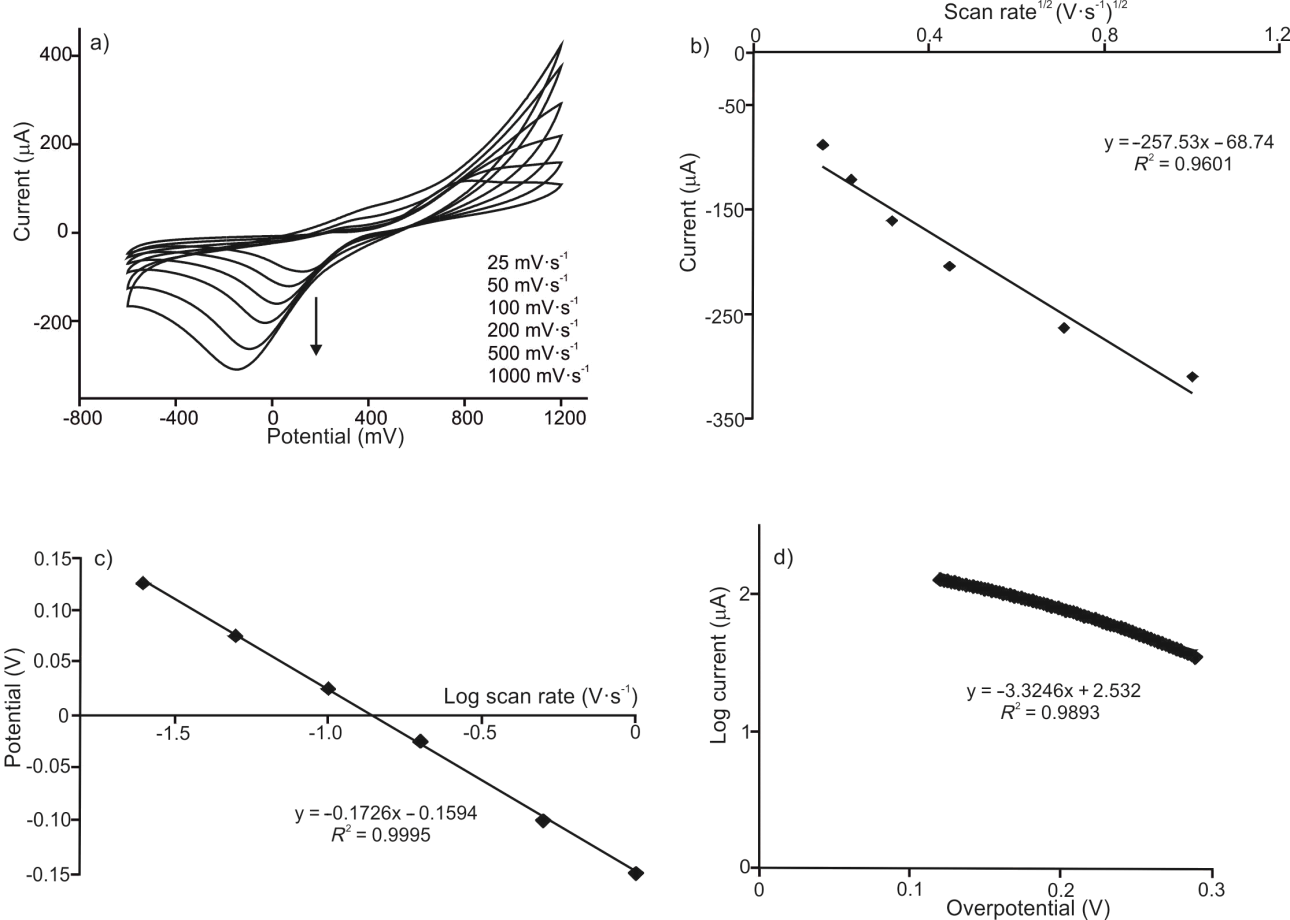
Effect of the scan rate in PEDOT/PSS/AuNP immersed in catechol 10^−3^ mol·L^−1^. (a) CVs collected at 0.025, 0.05, 0.1, 0.2, 0.5 and 1.0 V·s^−1^; (b) linear relationship between *I*_c_ and the square root of the scan rate; (c) linear relationship between *E*_c_ and the log of the scan rate and (d) Tafel plot representing the logarithm of the intensity vs the overpotential.

In order to further analyze the electron transfer and to evaluate the dynamic behavior of the electrodes, voltammograms were collected at different scan rates. The responses are illustrated in Figure 5 for the PEDOT/PSS/AuNP electrode immersed in catechol 10^−3^ mol·L^−1^. The cathodic peak potentials shifted to more negative potentials with increasing scan rate (Figure 5a). This suggests the involvement of a kinetic limitation between the electrode and the phenol. The cathodic peak currents (*I*_c_) varied linearly with the square root of the scan rate (ν^1/2^), demonstrating the diffusion-controlled nature of the electrode reaction (Figure 5b). The slopes and correlation coefficients for the measurement of all the sensors and biosensors immersed in catechol are collected in Table 3. The results obtained in hydroquinone are presented in Table 4. According to the slope values, the reduction of hydroquinone proceeds more rapidly than that of catechol. The slope values calculated in layered PEDOT/PSS/EM composites were higher than those observed in PEDOT/PSS, confirming the improvement of the charge transfer rates. It is also worth noting that the slopes found in enzymatic PEDOT/PSS/EM biosensors were higher than the values found in nonenzymatic PEDOT/PSS/EM electrodes. This result indicates that the charge transfer within the composite film and/or through the electrode interface is facilitated in biosensors and this improvement should be related with the enzymatic activity.

**Table 3:** Relationship between scan rate in sensors immersed in catechol 10^−3^ mol·L^−1^ calculated in the cathodic peak.

	*I*_c_ (μA) vs ν^1/2^ (V/s)^1/2^		*E*_c_ (V) vs log ν (V/s)			Log *I* (μA) vs η (V)		

Sensor	Slope	*R*^2^	Slope	*R*^2^	α*_n_*	Slope	*R*^2^	*n*

PEDOT/PSS	−115.71	0.946	−0.117	0.993	0.50	−4.536	0.998	1.87
PEDOT/PSS/LuPc_2_	−169.33	0.947	−0.144	0.994	0.41	−4.256	0.991	1.95
PEDOT/PSS/CuPc	−185.97	0.953	−0.135	0.997	0.44	−3.781	0.988	1.97
PEDOT/PSS/AuNP	−257.53	0.960	−0.173	0.999	0.34	−3.324	0.989	1.73
PEDOT/PSS-Tyr	−268.38	0.980	−0.169	0.985	0.35	−2.823	0.975	2.10
PEDOT/PSS/LuPc_2_-Tyr	−309.65	0.977	−0.184	0.992	0.32	−2.361	0.977	2.30
PEDOT/PSS/CuPc-Tyr	−230.25	0.981	−0.174	0.984	0.34	−2.632	0.968	2.20
PEDOT/PSS/AuNP-Tyr	−231.99	0.984	−0.181	0.984	0.33	−2.402	0.963	2.30

**Table 4:** Relationship between scan rate in sensors immersed in hydroquinone 10^−3^ mol·L^−1^ calculated in the cathodic peak.

	*I*_c_ (μA) vs ν^1/2^ (V/s)^1/2^		*E*_c_ (V) vs log ν (V/s)			Log *I* (μA) vs η (V)		

Sensor	Slope	*R*^2^	Slope	*R*^2^	α*_n_*	Slope	*R*^2^	*n*

PEDOT/PSS	−247.88	0.947	−0.168	0.992	0.50	−4.607	0.997	1.84
PEDOT/PSS/LuPc_2_	−299.33	0.954	−0.199	0.995	0.41	−3.247	0.999	2.10
PEDOT/PSS/CuPc	−270.75	0.967	−0.170	0.994	0.44	−3.680	0.996	2.02
PEDOT/PSS/AuNP	−300.56	0.966	−0.210	0.993	0.34	−3.391	0.997	1.70
PEDOT/PSS-Lac	−411.98	0.975	−0.143	0.988	0.41	−3.050	0.998	2.3
PEDOT/PSS/LuPc_2_-Lac	−511.99	0.978	−0.206	0.984	0.29	−2.004	0.999	2.4
PEDOT/PSS/CuPc-Lac	−532.85	0.968	−0.214	0.982	0.29	−2.003	0.999	2.4
PEDOT/PSS/AuNP-Lac	−463.63	0.969	−0.189	0.990	0.31	−2.254	0.999	2.3

In the scan rates ranging from 0.025 to 1 V·s^−1^, the cathodic peak potential (*E*_c_) showed a linear relationship with the logarithm of scan rate (log ν) (Figure 5c) according to Laviron’s equation (Equation 2) for a totally irreversible diffusion-controlled process [34–35].

[2]
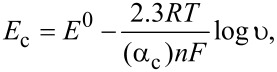


where α is the transfer coefficient, ν is the scan rate (expressed in V·s^−1^), *n* is the number of electrons involved in the rate-determining step, *R* is the ideal gas constant (8314 J·mol^−1^·K^−1^), *T* is the temperature (298 K) and *F* is Faraday's constant (95,484.56 C·mol^−1^).

The regression coefficients calculated for the analysis of all the sensors and biosensors immersed in catechol and hydroquinone are presented in Table 3 and Table 4, respectively.

The linear variation of the peak potential, *E*_c_, as a function of log ν suggests that the electrode process can be regarded as a totally irreversible reaction. From the slope of the graph, α*_n_* can be determined, where α is the transfer coefficient and *n* is the number of electrons transferred in the rate-determining step.

The slope indicated that the values α*_n_* have values between 0.3–0.5 for the cathodic peak. These values indicate the total irreversibility of the electron transfer process and also confirm the ideal diffusion-controlled mechanism [36].

The simplified Butler–Volmer equation can be applied to calculate the α values (Equation 3) [35]. The Tafel slope was calculated from the representation of log *I* vs the overpotential (η) obtained from a voltammogram collected in catechol or hydroquinone 10^−3^ mol·L^−1^ at a scan rate of 0.1 V·s^−1^ (Figure 5d). Then, using the results obtained from the Laviron equation for α*_n_*, the number of electrons transferred can be calculated.

[3]
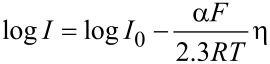


The calculated Tafel slopes, the α values and the number of electrons are listed in Table 3 and Table 4. As can be seen from these results, the Tafel slopes showed a less negative value in the electrodes modified with the composite PEDOT/PSS/EM. The values were even lower in the presence of an enzyme, confirming the improved electrocatalytic activity. This result further confirms that the combination of PEDOT/PSS with AuNPs and phthalocyanines can promote electron transfer in the active centers of biological molecules, increasing the relative activity of the enzymes.

The number of electrons involved in the reaction calculated from the Tafel slope and Equation 2 confirms a two electron process in all electrodes, confirming the proposed mechanism.

### Limit of detection

**Figure 6:**
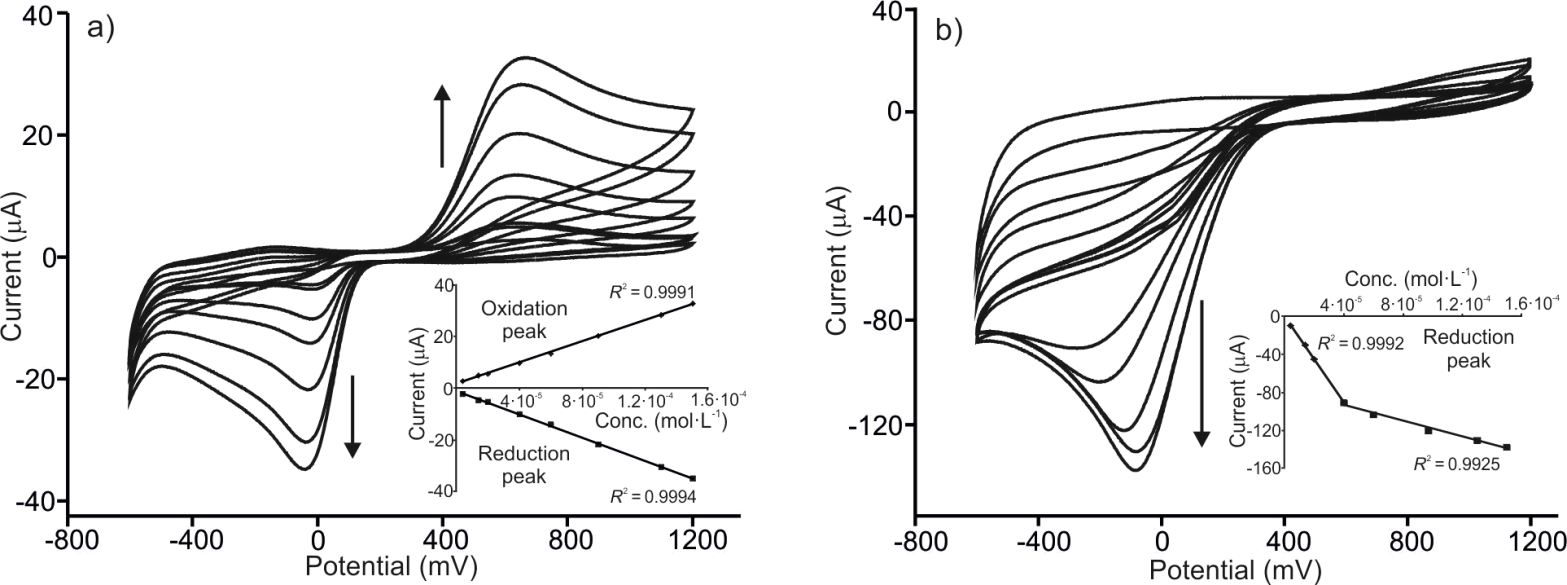
CVs of (a) PEDOT/PSS/LuPc_2_ and (b) PEDOT/PSS/LuPc_2_-Tyr immersed in increasing concentrations of catechol (from 4.0 × 10^−6^ mol·L^−1^ to 1.5 × 10^−4^ mol·L^−1^). Scan rate 0.1 V·s^−1^.

The limit of detection (LD) was evaluated by analyzing the response of the sensors towards phenol solutions with concentrations ranging from 4.0 × 10^−6^ mol·L^−1^ to 1.5 × 10^−4^ mol·L^−1^. The results are illustrated in Figure 6 for PEDOT/PSS/LuPc_2_ and PEDOT/PSS/LuPc_2_-Tyr immersed in catechol.

In PEDOT/PSS/EM electrodes, a linear relationship was observed between the current response signals and the concentration of catechol and hydroquinone, confirming that they may reliably be used for the determination of both compounds in this concentration range. The limit of detection calculated from the anodic peak (LD_A_) and the cathodic peak (LD_C_) (using the 3σ criteria) and correlation coefficients are collected in Table 5.

**Table 5:** Limit of detection and regression coefficients obtained for catechol and hydroquinone using the anodic (LD_A_) and the cathodic (LD_C_) peaks.

Sensor	LD_A_ (mol·L^−1^)	*R*^2^ (anode)	LD_C_ (mol·L^−1^ )	*R*^2^ (cathode)

Catechol				

PEDOT/PSS	4.96 × 10^−5^	0.993	6.58 × 10^−6^	0.998
PEDOT/PSS/LuPc_2_	1.23 × 10^−6^	0.999	1.03 × 10^−6^	0.999
PEDOT/PSS/CuPc	2.99 × 10^−6^	0.992	1.81 × 10^−6^	0.996
PEDOT/PSS/AuNP	2.18 × 10^−6^	0.998	0.87 × 10^−6^	0.999

Hydroquinone				

PEDOT/PSS	2.85 × 10^−5^	0.971	1.61 × 10^−5^	0.970
PEDOT/PSSLuPc_2_	1.80 × 10^−5^	0.996	9.41 × 10^−6^	0.997
PEDOT/PSS/CuPc	2.69 × 10^−5^	0.972	1.51 × 10^−5^	0.998
PEDOT/PSS/AuNP	1.97 × 10^−5^	0.993	7.55 × 10^−6^	0.996

The results confirmed that the LD towards catechol was improved almost one order of magnitude when using layered composite electrodes and attained the μmol·L^−1^ range. The improvement was not so marked in hydroquinone, but also in this case, the LD calculated from the cathodic peak was in the μmol·L^−1^ range.

The improved performance can be attributed to synergistic interactions between PEDOT/PSS and the additives similar to the interactions already described between PEDOT/PSS and graphene [37]. In our composites, PEDOT/PSS acts as the electron mediator and the additive further enhances the electron transfer due to the high electrical conductivity demonstrated in the impedance experiments.

In the case of PEDOT/PSS/EM enzymatic biosensors, the main amplification occurred in the cathodic wave. For this reason, the study of the LD was carried out only in the cathodic reduction peak.

When representing the peak current vs catechol concentration obtained from PEDOT/PSS/EM-Tyr, a linear relationship was obtained in the concentration range from 4.0 × 10^−6^ to 6 × 10^−5^ mol·L^−1^. Then, a change in the slope occurred and a new linear range was observed from 9.0 × 10^−5^ to 1.5 × 10^−4^ mol·L^−1^. The limit of detection calculated in both linear ranges is shown in Table 6. A LD in the low concentration range was attained (10^−7^ mol·L^−1^), that is, one order of magnitude lower than the LD obtained in the nonenzymatic sensor. The LD in the high concentration scale were similar to those obtained for the nonenzymatic sensors and were in the μmol·L^−1^ range. The obtained LDs were similar to values reported in recent works using tyrosinase and graphene as electron mediator [38–39]. The LD obtained for hydroquinone was also one order of magnitude lower than in nonenzymatic sensors and attained values of 10^−6^ mol·L^−1^.

**Table 6:** Limit of detection and regression coefficients obtained for catechol and hydroquinone using the cathodic (LD_C_) peaks using biosensors.

Sensor	LD_C_ (mol·L^−1^)		*R*^2^ (cathode)	

Conc. range	4 × 10^6^–6 × 10^−5^	9 × 10^−5^–1.5 × 10^−4^	4 × 10^−6^–6 × 10^−5^	9 × 10^−5^–1.5 × 10^−4^

Catechol				

PEDOT/PSS-Tyr	6.62 × 10^−7^	6.55 × 10^−6^	0.9862	0.987
PEDOT/PSS/LuPc_2_-Tyr	4.62 × 10^−7^	2.53 × 10^−6^	0.9925	0.999
PEDOT/PSS/CuPc-Tyr	4.37 × 10^−7^	3.16 × 10^−6^	0.9971	0.936
PEDOT/PSS/AuNP-Tyr	3.88 × 10^−7^	2.80 × 10^−6^	0.9889	0.971

Hydroquinone				

PEDOT/PSS-Lac	6.00 × 10^−6^		0.997	
PEDOT/PSS/LuPc_2_-Lac	1.11 × 10^−6^		0.996	
PEDOT/PSS/CuPc-Lac	1.52 × 10^−6^		0.999	
PEDOT/PSS/AuNP-Lac	2.51 × 10^−6^		0.998	

## Conclusion

Owing to their unique electrochemical properties, layered composite PEDOT/PSS/EM and PEDOT/PSS/EM-Enz electrodes offer efficient electron transfer, low detection limit, high sensitivity and good reproducibility toward the oxidation of catechol and hydroquinone.

Furthermore, the combination of PEDOT with AuNPs or phthalocyanines can promote electron transfer in the active centers of biological molecules, increasing the relative activity of the enzymes. The linear relationship between the peak current and the square root of the scan rate for both PEDOT/PSS/EM sensors and PEDOT/PSS/EM-Enz biosensors in catechol and hydroquinone indicate a diffusion-controlled analyte electrocatalytic oxidation, and Tafel slopes confirmed the improved electrocatalytic activity. The modified electrodes designed here show excellent limits of detection that are comparable with those found in literature. The reported sensors could be used to analyze the phenolic content of foods and beverages such as wine, must, coffee, saffron, etc.

## Experimental

### Reagents and solutions

Poly(3,4-ethylenedioxythiophene)/poly(styrene sulfonate) (PEDOT/PSS) aqueous solution (3.0–4.0% in H_2_O, high conductivity grade) and copper(II) phthalocyanine tetrasulfonic acid tetrasodium salt (CuPc, 0.05 g/L) were purchased from Sigma-Aldrich. Gold nanoparticle (AuNPs, 30–40 nm) colloids were synthesized according to a modification of the procedure proposed by Slot and Geuze [12,40]. Using this procedure, a red colloid with a UV absorbance maximum at λ = 540 nm was obtained. The lutetium(III) bisphthalocyaninate (LuPc_2_, 0.05 g/L) was synthesized following a previously published procedure [41].

Phenol oxidase enzymes were purchased from Sigma-Aldrich: tyrosinase (from mushroom, activity ≥1000 U·mg^−1^) and laccase (from *Trametes versicolor*, activity ≥10 U·mg^−1^). 5 mg·L^−1^ solutions of tyrosinase and laccase were prepared in buffered phosphate 0.01 mol·L^−1^ (pH 7.0).

### Preparation of the electrochemical sensors and biosensors

Sensors were prepared using a spin coater, model 1H-D7 (Micasa Co., Tokyo, Japan). ITO glass substrates (1 cm^2^ surface area) were used as the substrate. Prior to the film deposition, the substrates were washed in an ultrasonic bath with acetone and rinsed twice with deionized water (MilliQ).

PEDOT/PSS was diluted 1:10 in deionized water and stirred in an ultrasonic bath for 10 min. Then, 100 μL of the solution was dropped onto the ITO glass and spin-coated at 2000 rpm for 120 s (slope of 120 s). The PEDOT/PSS films obtained were annealed at 150 °C for 15 min.

PEDOT/PSS/EM layered sensors were prepared on the surface of PEDOT/PSS films by spin coating 200 μL of CuPc and AuNPs, or 100 μL of LuPc_2_ at 2000 rpm for 120 s (slope of 120 s), followed by annealing at 150 °C for 15 min.

PEDOT/PSS/EM enzymatic biosensors were prepared by depositing laccase or tyrosinase onto the PEDOT/PSS/EM sensors. For this purpose, 50 μL of 0.01 mol·L^−1^ phosphate buffer (pH 7.0) containing 5 mg·mL^−1^ of enzyme were deposited onto the PEDOT/PSS/EM electrode. After drying at room temperature for approximately 45 min., the biosensors were immersed in glutaraldehyde (2.5% v/v, buffer solution) for 5 min and dried in air for 15 min at room temperature. The biosensors were then rinsed with phosphate buffer to remove any unbound enzyme and stored at 4 °C.

### Characterization of the sensors

Scanning electron microscopy (SEM) (FEI, QUANTA 200F) was used to record the images of the electrode surfaces. A square resistance was measured using a four-point tester (HAAMEG, HM 8040-2).

Electrochemical impedance spectroscopy (EIS) experiments were performed using a Solartron impedance analyzer. The measurements were carried out by applying a signal amplitude of 10 mV, at a working potential of −0.5 V with frequencies varied logarithmically from 0.1 Hz to 100 kHz. The measurements were made by immersing the electrode in 10^−3^ mol·L^−1^ catechol in 0.01 buffer phosphate (pH 7). The impedance spectra were fitted with the aid of the Zview2 software.

Voltammetric measurements were obtained using a potentiostat/galvanostat PGSTAT128 (Autolab Metrohm, Utrecht, Netherlands). The electrochemical cell was a three-electrode system using the corresponding PEDOT/PSS, PEDOT/PSS/EM or PEDOT/PSS/EM-Enz modified electrode as the working electrode; the reference electrode was Ag|AgCl/KCl 3 mol/L and the counter electrode was a platinum sheet with a surface area of 1 cm^2^.

Cyclic voltammetry was carried out from −0.6 V to +1.2 V (vs Ag/AgCl) with a scan rate of 0.1 V·s^−1^, except when indicated otherwise. A phosphate buffer solution (0.01 mol·L^−1^; pH 7.0) was employed as the electrolytic medium in electroanalysis experiments**.** The influence of the potential sweep rate was studied in 10^−3^ mol·L^−1^ catechol or 10^−3^ mol·L^−1^ hydroquinone in 0.01 phosphate buffer (pH 7), while varying the scan rates from 0.025 to 1.0 V·s^−1^.

The limits of detection (LD) was calculated from peak current responses taken from voltammograms recorded at different concentrations from 4·10^−6^–1.5·10^−4^ mol/L, following the “3sd/m” criterion, where “m” is the slope of the calibration graph and “sd” was estimated as the standard deviation (*n* = 5) of the voltammetric signal at the concentration level corresponding to the lowest concentration of the calibration plot.
